# IPF-Fibroblast Erk1/2 Activity Is Independent from microRNA Cluster 17-92 but Can Be Inhibited by Treprostinil through DUSP1

**DOI:** 10.3390/cells10112836

**Published:** 2021-10-21

**Authors:** Sabrina Blumer, Lei Fang, Wei-Chih Chen, Petra Khan, Katrin Hostettler, Michael Tamm, Michael Roth, Christopher Lambers

**Affiliations:** 1 Pulmonary Cell Research & Pneumology, Department of Biomedicine & Internal Medicine, University Hospital Basel, Petersgraben 4, CH-4031 Basel, Switzerland; sabrina.blumer@unibas.ch (S.B.); lei.fang@usb.ch (L.F.); wiji.chen@gmail.com (W.-C.C.); petra.khan@unibas.ch (P.K.); katrin.hostettler@usb.ch (K.H.); Michael.tamm@usb.ch (M.T.); 2 Department of Chest Medicine, Taipei Veterans General Hospital, Taipei 11217, Taiwan; 3 School of Medicine, Faculty of Medicine, National Yang Ming Chiao Tung University, Taipei 11266, Taiwan; 4 Institute of Emergency and Critical Care Medicine, National Yang Ming Chiao Tung University, Taipei 11266, Taiwan; 5 Thoracic Surgery, University Hospital Vienna, Währinger Gürtel 10-14, A-1090 Vienna, Austria; christopher.lambers@meduniwien.ac.at

**Keywords:** idiopathic pulmonary fibrosis, fibroblast, transforming growth factor β1, platelet-derived growth factor-BB, Erk1/2 mitogen-activated protein kinase, dual specificity phosphatase 1, proliferation, microRNA cluster 17-92

## Abstract

Idiopathic pulmonary fibrosis (IPF) is a progressive terminal lung disease, and therapies aim to block fibrosis. Fibroblast proliferation is controlled by C/EBP-β, microRNA cluster 17-92 (miR17-92), and Erk1/2 mitogen-activated protein kinase. This study assessed the role of miR17-92 in IPF-fibroblast proliferation and its modification by treprostinil. Fibroblasts were isolated from eight IPF patients, five interstitial lung fibrosis patients, and seven control lungs. Fibroblasts were stimulated with TGF-β1 over 24 h. The miR17-92 expression was analyzed by RT-qPCR, and protein expression by Western blotting. TGF-β1 upregulated C/EBP-β in all fibroblasts, which was reduced by treprostinil in control-fibroblasts, but not in IPF-fibroblasts. Compared to controls, the guide strands miR-19a-3p, miR-19b-3p, miR-20a-5p, and miR-92a-3p, as well as the passenger strands miR-17-3p, miR-18-3p, miR-19a-1-5p, and miR-92a-5p were significantly increased in IPF-fibroblasts. In controls, TGF-β1 and treprostinil significantly reduced specific miR17-92 members. IPF-fibroblast proliferation was inhibited by treprostinil through increased expression of the Erk1/2 inhibitor DUSP1. These data suggest that proliferation control via miR17-92 and C/EBP-β is disrupted in IPF-fibroblasts. Therefore, the inhibition of early stages of signaling cascades or specific mitogen receptors might be less effective. However, the increased proliferation is sensitive to Erk1/2 inhibition by treprostinil-induced DUSP1.

## 1. Introduction

Idiopathic pulmonary fibrosis (IPF) is a chronic progressive inflammatory lung disease resulting in the formation of scarred alveoli and loss of function [1]. The cause of IPF is not known, but air pollution, inorganic dust, organic dust, cigarette smoke, viral infections, or genetic predisposition have been suggested to trigger lung scarring or may increase the risk of IPF [2]. The survival of IPF patients is poor, and the limited therapeutic options include pharmaceutical compounds and lung transplantation [3,4]. Therefore, new targets for IPF therapy have to be identified, which requires a better understanding of the etiology and pathogenesis. 

IPF is characterized by epithelium dysfunction, as well as fibroblast hyperplasia and their transformation into myo-fibroblasts [5]. These IPF pathologies were linked to the increased expression of transforming growth factor-β1 (TGF-β1) that was highly expressed in tissue and lung fluids of IPF patients [6]. Suppression of TGF-β1 signaling by tranilast significantly reduced the deposition of collagen and fibronectin in a mouse model of IPF [7]. TGF-β1 can be induced by bleomycin and contributes to the etiology of IPF by inducing epithelial to mesenchymal transition (EMT), reducing differentiation and apoptosis, as well as increasing fibroblast proliferation [8,9]. In human lung fibroblasts, platelet-derived growth factor (PDGF)-BB increased proliferation through the expression of TGF-β1 and subsequent activation of Erk1/2 mitogen-activated protein kinase (MAPK) [10,11]. A negative regulatory mechanism of Erk1/2 MAPK are microRNAs (miRs), including the miR cluster 17-92 (miR17-92) in human lung fibroblasts [12] and IPF-derived fibroblasts [13].

Regarding the role of other miRs in IPF, modified expression of 22 different miRs were reported [14]. However, different studies could not conclude that a specific set of miR was linked to the etiology of IPF. In human IPF lungs and in a mouse model, the expression of some miRs from miR17-92 was significantly reduced due to DNA methylation of the promoter region [15]. In other chronic conditions, down-regulation of specific miR17-92 members was linked to increased expression of Erk1/2 MAPK, PRMT1, and proliferation [12]. However, the role of miR17-92 in the pathogenesis of IPF was not investigated. In IPF-derived human fibroblasts and tissue protein, arginine methyltransferase 1 (PRMT1) was up-regulated and mediated cell proliferation [16]. In other cell types, PRMT1 expression was controlled by cyclic AMP (cAMP) [17,18], suggesting that cAMP-stimulating drugs might regulate miR17-92 and PRMT1 in IPF.

Prostacyclins increased intracellular cAMP and therefore should affect the above described fibroblast-proliferation-controlling mechanism. In this context, it is worthwhile to note that besides established IPF therapies, the INCREASE study demonstrated that inhaled treprostinil improved forced vital capacity (FVC) in patients with interstitial lung diseases, compared to placebo [19]. Therefore, we assessed the expression and regulation of the miR17-92 members and their regulation by TGF-β1 in the presence and absence of the cAMP activator, treprostinil, in fibroblasts obtained from healthy lung or lung transplant recipients, with interstitial lung fibrosis or IPF.

## 2. Materials and Methods

### 2.1. Study Cohort

Lung tissues were provided by the Department of Thoracic Surgery (University Hospital Vienna, Austria). The primary cell lines used in this study were established between 2015 and 2018. Patients with proven or suspected auto-immune disorders were excluded from the study. Non-fibrotic control cells were obtained from patients undergoing lung surgery for other reasons. 

Fibrotic lung diseases were diagnosed as Category C in accordance with the Eurotransplant classification [20] and the ATS/ERS classification [21]. IPF was defined by radiology and histology. The term “interstitial fibrosis” describes patients for whom the CT-scan and the histology were inconsistent with the diagnosis UIP pattern (IPF). Neither patients with connective tissue disease, nor auto-immune disorders, were included in this study. Details of the probands are shown in Table 1. All patients had end-stage interstitial lung diseases, and were scheduled for transplantation.

### 2.2. Cell Generation

Fibroblasts were isolated from peripheral lung tissues by selective medium (CnT-PR-F, CellnTec, Bern, Switzerland) over a period of 7–14 days. Fibroblasts were propagated in RPMI-1640 supplemented with 10% fetal calf serum, 20 mM HEPES, 1× essential amino acid mix, and 8 mM L-glutamax (all Gibco/BRL, Baar, Switzerland). Cells were characterized as described earlier by immunohistochemistry [10,11].

For experiments, fibroblasts were incubated for 2 days in CnT-PR-F medium, before being stimulated with either TGF-β1 (10 ng/mL, R&D System, Abington, UK) or treprostinil (10 μM, United Therapeutics Corporation, Research Triangle Park, Silver Spring, NC, USA), based on earlier studies [10,11]. 

For DUSP1 inhibition, sub-confluent cells (10,000/cm^2^) were pre-incubated with the DUSP1 inhibitor BCI-CAS 15982-84-0 as advised by the distributor (cat: 317496, Calbiochem/Merck, Schaffhausen, Switzerland), followed by treprostinil (10 µM, 30 min) and TGF-β1 (10 ng/mL, 48 h) treatment. Cell counts were performed manually using a Neubauer chamber slide.

### 2.3. Western Blotting

Confluent cell layers were serum deprived overnight before being stimulated with TGF-β1 (10 ng/mL) in the presence or absence of treprostinil (10 µM). Pre-incubation with treprostinil was 30 min. Fibroblasts were lysed in RIPA buffer (#R0278, Sigma-Aldrich, Buchs, Switzerland) containing protease inhibitors cocktail (#78447, ThermoFisher Scientific Inc., Waltham, MA, USA) and quantified by BCA protein analysis kit (#23227, ThermoFisher Sci). Equal amounts of denatured protein (20 μg) were size-fractionated in 4–12% SDS–PAGE (#M41212, GeneScript, Leiden, The Netherlands) and transferred onto nitrocellulose membranes (#88018, ThemoFisher Sci). Proteins were detected by specific antibodies as listed in the Supplementary Material, including GAPDH (#2118), C/EBP-β (#3087), t-ERK1/2 (#9102), p-ERK1/2 (#4370, all: Cell Signaling Technology, Bioconcept, Allschwil, Switzerland), and DUSP1 (#ab195261, Abcam, Cambridge, UK), followed by species matched Horse Radish Peroxide (HRP)-labelled secondary antibodies. Protein bands were visualized by HRP and Azure C300 digital imaging system (Axonlab, Baden, Switzerland) and further analyzed by software ImageJ (ImageJ Java v1.8.0_172, NIH, Bethesda, MD, USA) as described earlier [22].

Real-time quantitative PCR: For detection of miRs, total RNA was isolated from primary fibroblasts derived from control, IPF, and fibrosis patients using the NucleoSpin miRNA Kit (Macherey-Nagel, Düren, Germany). Then, 1 µg of RNA was used to generate first strand complementary DNA (cDNA) with miR-specific stem loop reverse transcription reaction using the Mir-X miRNA First-Strand Synthesis Kit (Takara Bio Europe, Kusatsu, Japan). The cDNA was diluted 1:20 before usage and amplified on a ViiA 7 Real-Time PCR system (ThermoFisher Sci) with FastStart Universal SYBR Green PCR Master Mix (Sigma-Aldrich) on a 384-well plate. The data was analyzed and quantified using the ∆∆-Ct method, and the purity of PCR products was confirmed by using melting curve analysis. U6 small nuclear RNA was used as a housekeeping gene, as well as for normalization to miRs expression. 

The following primers for the members of the human miR17-92 were used: hsa-miR-17-5p: CAA AGT GCT TAC AGT GCA GGT AG, hsa-miR-17-3p: ACT GCA GTG AAG GCA CTT GTA G, hsa-miR-18a-5p: TAA GGT GCA TCT AGT GCA GAT AG, hsa-miR-18a-3p: ACT GCC CTA AGT GCT CCT TCT GG, hsa-miR-19a-5p: AGT TTT GCA TAG TTG CAC TAC A, hsa-miR19a-3p: TGT GCA AAT CTA TGC AAA ACT GA, hsa-miR-20a-5p: TAA AGT GCT TAT AGT GCA GGT AG, hsa-miR20a-3p: ACT GCA TTA TGA GCA CTT AAA G, hsa-miR-19b-1-5p: AGT TTT GCA GGT TTG CAT CCA GC, hsa-miR-19b-1-3p: TGT GCA AAT CCA TGC AAA ACT GA, hsa-miR-92a-5p: AGG TTG GGA TCG GTT GCA ATG CT, hsa-miR-92a-3p: TAT TGC ACT TGT CCC GGC CTG T, hsa-miR-21-5p: TAG CTT ATC AGA CTG ATG TTG A.

The miR17-92 promoter isolation and sequencing: Genomic DNA was isolated from fibroblasts of controls and IPF patients, but not from patients with interstitial lung disease. The miR17-92 promoter region containing the two putative C/EBP-β binding sites were amplified with specific primers (Table S1) by PCR. Sanger sequencing was performed by Microsynth AG (Basel, Switzerland).

Genomic DNA was isolated from control-fibroblasts, and then specific primers (Table S1) were used for PCR to generate six different miR17-92 promoter sequences (Figure S1). These promoter sequences were cloned in front of a Luciferase reporter gene (pCLuc-Basic 2.0 vector, Addgene, Watertown, MA, USA). Fibroblasts from both IPF patients and controls were seeded into 6 well-plates at 70% density and co-transfected with 0.3 µg luciferase reporter vector and 0.1 µg red fluorescence protein scramble shRNA before being stimulated (Figure S1). Transfection with empty pCluc-Basic 2.0 vector served as negative control. Luciferase reporter assay was performed according to the manufacturer’s instructions.

### 2.4. Transfection

Fibroblasts were transiently transfected using HiPerfect Reagent Kit (#301705, Qiagen, Hombrechtikon, Switzerland) according to the manufacturer’s instructions. Synthetic miR-19a-3p mimic and miR-19a-3p inhibitor were also from Qiagen (#MSY0000073, #MIN0000073). Cells were seeded into 6-well plates at 70% confluence. Each well was treated with transfection solution as follows: 100 µL serum-free medium was mixed with 6 µL HiPerfect and 50 nM of one of the miRs. The solution was vortexed and kept at room temperature for 4 min before being added to a cell culture well, and 1 mL of cell culture medium was added dropwise. After 48 h, the medium was replaced, and the cells were ready for subsequent experiments.

### 2.5. Luciferase Reporters

Different miR17-92 promoter constructs were transfected into semi-confluent cells using Effectene Transfection Reagent Kit (#031425, Qiagen) followed by stimulation either with PDGF-BB (1 ng/mL, R&D Systems, Abingdon, UK) or FA (1 ng/mL, Sigma Aldrich, Basel, Switzerland). Negative control cells were co-transfected with an empty pCLuc-Basic 2.0 vector. Red fluorescence dye from the shRNA scramble were visualized under live-cell fluorescence microscope (Witec, Sursee, Switzerland) (Figure S1) and indicated approximately 30% transfection rates. After 48 h, post-transfection and stimulation, the luciferase reporter assay was performed with the Biolux Cypridina Luciferase Assay kit (#E3309, New England Biolabs/BioConcept, Ipswich, MA, USA) according to the manufacturer’s instructions.

### 2.6. Proliferation

Proliferation was determined by manual cell count using a Neubauer hemocytometer. Fibroblasts were seeded at 80% confluence (10,000 cells per cm^2^ in six-well plate (Sarstedt, Sevelen, Switzerland) and allowed to adhere overnight in growth medium. Fibroblasts were serum deprived overnight before being stimulated in the presence or absence of treprostinil over 48 h. Fibroblasts were harvested by trypsinization and counted.

### 2.7. Statistics

The null hypothesis was that the expression of miR17-92 was not different in fibroblasts obtained from controls or fibrotic lungs, and that it was not affected by TGF-β1 or treprostinil treatment. Statistical analysis was performed using GraphPad-Prism7 software. The RT-qPCR data was normalized to U6 snRNA and is presented as mean (±SEM). In order to compare the fibroblasts from controls to those of patients with IPF or interstitial lung fibrosis, an unpaired Student’s *t*-test followed by a Mann–Whitney unpaired U-test were applied. For comparison within one patient group, a paired Student’s *t*-test followed by a Wilcoxon test were used. *p*-values < 0.05 were considered being statistically significant.

## 3. Results

### 3.1. Disease-Specific Expression and Response of miR17-92Members in IPF-Fibroblasts

**Figure 1:**
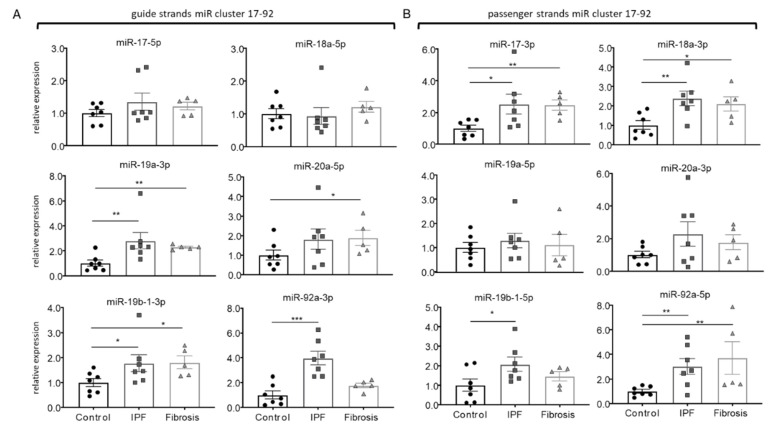
Disease-specific expression of miR17-92 members in isolated human primary cells. (**A**) Relative expression of guide strands miR17-92 in mesenchymal cells isolated from controls (*n* = 7), idiopathic pulmonary fibrosis (IPF) patients (*n* = 7), and interstitial lung fibrosis (Fibrosis) (*n* = 5). (**B**) Relative expression of passenger strands miR17-92 in mesenchymal cells isolated from controls (*n* = 7), IPF patients (*n* = 7), and interstitial lung fibrosis (*n* = 5). Bars represent mean ± SEM. * indicates *p* < 0.05, ** indicates *p* < 0.01, *** indicates *p* < 0.001.

Comparing the expression of the miR17-92 members between controls (*n* = 7), interstitial lung fibrosis (*n* = 5), and IPF-derived fibroblasts (*n* = 8), the expression of miR-19a-3p and miR-19b-1-3p were significantly increased in the two fibrosis groups, while miR-20a-5p was only upregulated in lung fibrosis, and miR-92a-3p was specifically upregulated in IPF-fibroblasts (Figure 1A). A similar disease-specific expression pattern was observed for the passenger strands, which were upregulated in both fibrotic groups for miR-17-3p, miR-18a-3p, and miR-92a-5p (Figure 1B). The expression of miR-19b-1-5p was only significantly upregulated in IPF-fibroblasts (Figure 1B).

The regulation of the miR17-92 was further investigated by treating the fibroblasts with either TGF-β1 (10 ng/mL), or treprostinil (10^−8^ M), or a sequential treatment with pre-incubation of 30 min with treprostinil, followed by TGF-β1. The expression level of miR17-92 members was determined after 24 h. 

**Figure 2:**
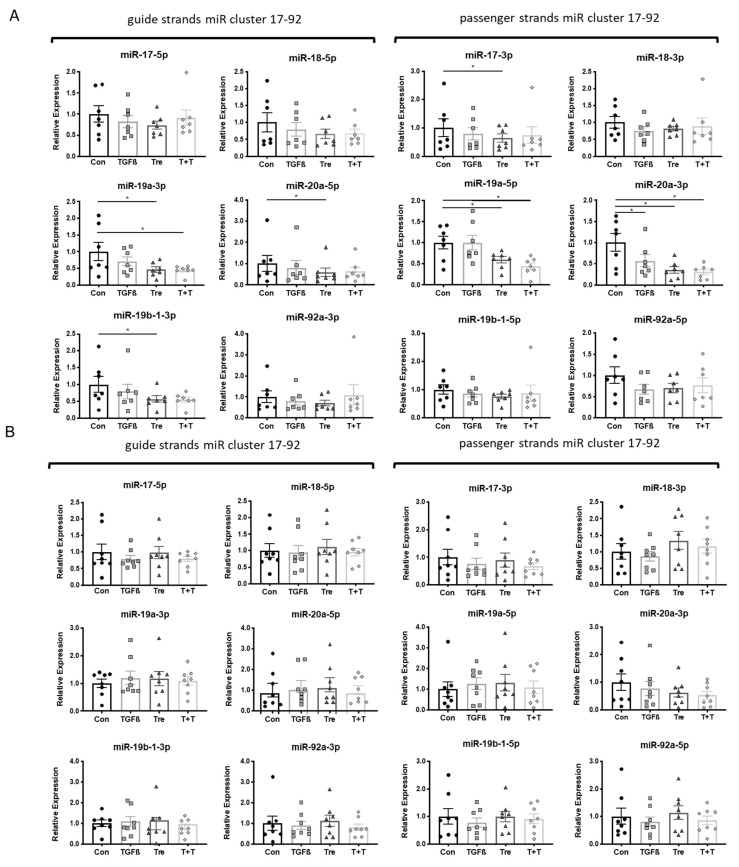

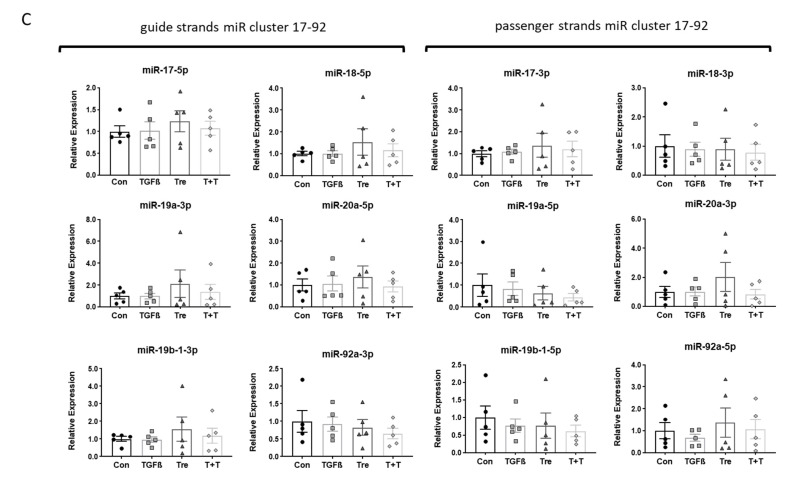
Disease-specific modification of miR17-92 expression by TGF-β1 and treprostinil. Cells were treated with either TGF-β1 (10 ng/mL), or treprostinil (10^−8^ M) alone, or combination of both (T + T). MiR expression was determined after 24 h for guide and passenger strands. Relative expression of miR17-92 in (**A**) cells of controls (*n* = 7); (**B**) IPF patients (*n* = 7); and (**C**) interstitial lung fibrosis patients (*n* = 5). Bars represent mean ± SEM. * indicates *p* < 0.05. TGFβ: TGF-β1 (10 ng/mL); Tre: treprostinil (1 × 10^−8^ M); T+T: TGF-β1 (10 ng/mL) plus treprostinil (1 × 10^−8^ M).

Stimulating with either TGF-β1 or treprostinil revealed that the fibroblasts from the two fibrosis groups were not responsive to either treatment, while control fibroblasts showed a stimulus and miR-specific downregulation as described below. TGF-β1 significantly reduced the expression of miR-20a-3p, while treprostinil significantly reduced the expression of miR-19a-3p, miR-19a-5p, and miR-20a-3p (Figure 2A). In addition, when combined, TGF-β1 and treprostinil downregulated miR-19a-3p, miR-19a-5p, and miR-20a-3p (Figure 2A). The expression of the different miR17-92 members in fibroblasts obtained from patients with either IPF or interstitial lung fibrosis is shown in Figure 2B,C.

### 3.2. MiR Inhibition

**Figure 3:**
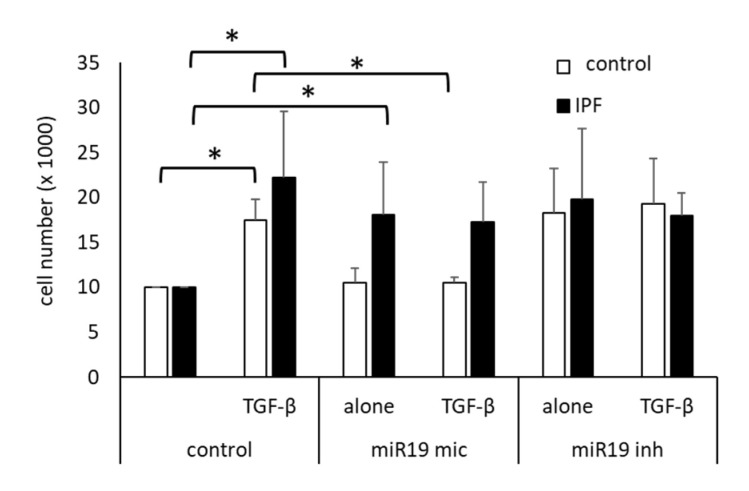
Modification of miR-19a-3p on cell proliferation. Proliferation was determined 2 days after stimulation with 10 ng/mL TGF-β1 (TGF-β), and in non-treated cells. Prior to the stimulation, cells were transfected with either a mimic of miR19a-3p (miR19 mic) or an inhibitor of miR-19a-3p (miR19 inh). Bars represent the mean ± SEM of cell counts obtained in 5 control fibroblast lines and of 5 IPF cell lines. *p*-values were calculated by Student’s *t*-test (2 sided paired): * indicates *p* < 0.05. TGF-β: TGFβ1 (10 ng/mL); miR-19a-3p mimic vector: miR19; miR-19a-3p inhibitor: miR19 inh.

To assess the role of miR17-92 members on cell proliferation, cells were transfected with a synthetic miR-19a-3p mimic vector or a miR-19a-3p inhibitor. As shown in Figure 3, TGF-β1-induced cell proliferation increased significantly over 2 days. The TGF-β1-induced proliferation was significantly reduced in control cells by miR19 mimic, but in IPF cells, miR19 mic had no significant effect (Figure 3). The miR19 inhibitor had a proliferative effect on untreated cells, but did not further increase proliferation induced by TGF-β1 (Figure 3). 

### 3.3. Disease-Specific Loss of miR-21a-5p Response

**Figure 4:**
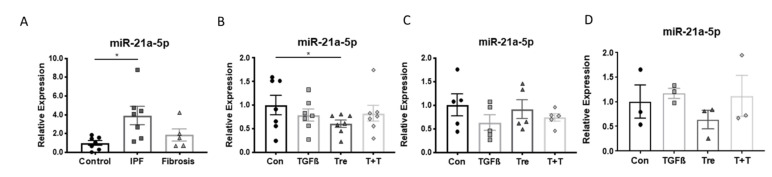
Disease-specific expression of miR-21a-5p and its modification by TGF-β1 and treprostinil. (**A**) MiR expression was determined in cells of controls (*n* = 7), IPF patients (*n* = 7), and interstitial lung fibrosis (*n* = 5). (**B**) Modification of miR-21a-5p by TGF-β1 and treprostinil in control cells, (**C**) in IPF cells, and (**D**) in interstitial lung fibrosis cells. Bars represent mean ± SEM. * indicates *p* < 0.05. TGFβ: TGF-β1 (10 ng/mL); Tre: treprostinil (1 × 10^−8^ M); T+T: TGF-β1 (10 ng/mL) plus treprostinil (1 × 10^−8^ M).

In addition to miR17-92, the expression of miR-21a-5p was assessed in the three fibroblast groups. The expression of miR-21-5p was significantly higher in IPF-fibroblasts compared to controls, while fibrosis fibroblasts were in the range of controls, with one exception (Figure 4A). 

The expression level of miR-21a-5p was assessed in cells of the three patient groups after treatments with either TGF-β1 (10 ng/mL), or treprostinil (10^−8^ M), or a sequential treatment with pre-incubation of 30 min with treprostinil, followed by TGF-β1. In control cells, treprostinil significantly reduced the expression of miR-21a-5p, but TGF-β1 had no effect (Figure 4B). None of the treatments significantly reduced the expression of miR-21a-5p in fibroblasts obtained from patients with IPF (Figure 4C) and fibrotic lung diseases (Figure 4D).

The human promoter of the miR17-92 was isolated from genomic DNA obtained from IPF patients (*n* = 4) and non-fibrotic controls (*n* = 5) as shown in Figure S1A. 

In order to follow the cause of the reduced promoter response in IPF-derived fibroblasts (*n* = 4), the sequence of the two C/EBP binding regions was analyzed by Sanger method and compared to the sequence from non-IPF controls (*n* = 5). As shown in Figure S1A, the sequence of the two C/EBP binding promoter regions in individual patients was compared to the published human genomic sequence and showed no difference. 

Due to the length of the promoter, it was divided into three sections, which were assigned A, B, and C (Supplementary Figure S1B). Each section was inserted in front of a luciferase reporter gene, and fibroblasts were transiently transfected before being stimulated with platelet-derived growth factor (PDGF)-BB for up to 48 h. As shown in Figure S1B, only those reporter constructs including insert C expressed luciferase activity. It should also be noted that the response of IPF-derived fibroblasts was lower than that of non-fibrotic fibroblasts (Figure S1B). Folic acid (FA) was used as a known stimulator for miR17-92 [23]. FA, but not PDGF-BB stimulation up-regulated luciferase activity in non-IPF-fibroblasts (Figure S1B). 

### 3.4. Proliferation Control of IPF-Fibroblasts Is Reduced by Treprostinil-Dependent DUSP1 Inhibition of Erk1/2

**Figure 5:**
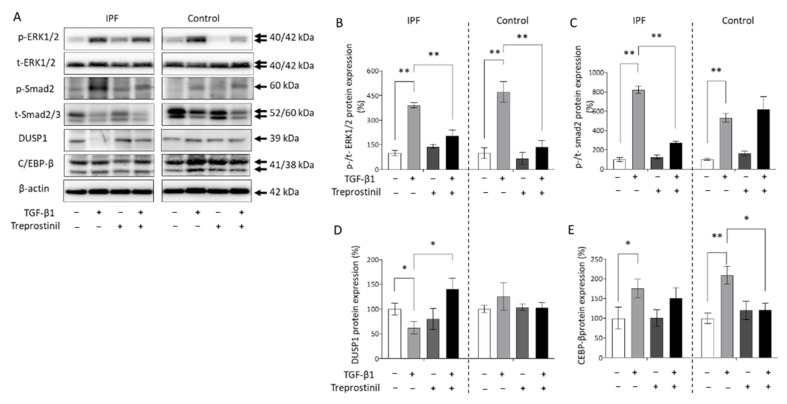
The effect of treprostinil and TGF-β1-induced signaling and proliferation in control and IPF cells. (**A**) Representative Western blots obtained in cells isolated from IPF patients or controls. (**B**) Image analysis of Western blots (*n* = 3) for the activation of Erk1/2 MAPK, (**C**) Smad2, (**D**) DUSP1, and (**E**) C/EBP-β. Bars represent mean ± SEM. * indicates *p* < 0.05, ** indicates *p* < 0.01. TGF-β1 at 10 ng/mL, treprostinil at 1 × 10^−8^ M.

Cell proliferation induced by TGF-β1 or PDGF-BB is controlled by the activation of Erk1/2 MAPK, Smad2/3, and C/EBP-β. Therefore, we compared the effect of treatment with TGF-β1 in the presence and absence of treprostinil (10^−8^ M) on the expression and activation of these proteins as shown in Figure 5A. Protein expression was analyzed by Image-J based on the grey-scale of the Western blot bands (Figure 5B–E). Comparing the baseline expression of Erk1/2 MAPK, we did not observe a disease-specific upregulation (Figure 5A). The phosphorylation of Erk1/2 MAPK induced by TGF-β1 was significantly reduced after pre-incubation with treprostinil as shown by Western blotting and image analysis in Figure 5B. The TGF-β1-induced activation of Smad2 was only reduced in IPF-derived fibroblasts by treprostinil, but not in control fibroblasts (Figure 5C). The activity of Erk1/2 MAPK is regulated by its inhibitor DUSP1, which was reduced by TGF-β1 in IPF, but not in control fibroblasts (Figure 5D). Pre-treatment with treprostinil significantly upregulated the expression of DUSP1 and thereby counteracted the effect of TGF-β1 in IPF fibroblasts (Figure 5D). Furthermore, TGF-β1 upregulated the protein expression of C/EBP-β, which is known as a negative regulator of miR17-92. This might explain the reduced expression of some cluster members shown in Figure 1. Pre-incubation with treprostinil significantly prevented the TGF-β1-induced C/EBP-β expression in control fibroblasts, but not in IPF-fibroblasts (Figure 5E). 

**Figure 6:**
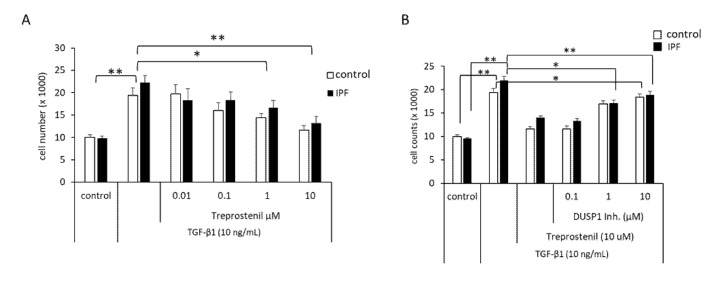
Proliferation control by treprostinil and DUSP1. (**A**) Cell counts were performed 48 h after stimulation with TGF-β1 (10 ng/mL) in the absence or presence of treprostinil at increasing concentrations. (**B**) Cells were pre-incubated (30 min) with increasing concentrations of the DUSP1 inhibitor BCI-CAS 15982-84-0 (DUSP1 Inh.) before being treated with treprostinil (10 µM, 30 min), followed by stimulation with TGF-β1 (10 ng/mL). Bars represent mean ± SEM of five control lines or eight IPF cell lines, respectively. *p*-values were calculated by ANOVA and Student’s *t*-test. * indicates *p* < 0.05, ** indicates *p*< 0.01.

Finally, the effect of treprostinil on cell proliferation and the role of DUSP1 was assessed in control- and IPF-fibroblasts. As shown in Figure 6A, TGF-β1-induced proliferation was downregulated by treprostinil in a concentration-dependent manner in both control and IPF cells. The activation of DUSP1 by treprostinil was blocked by 30 min pre-incubation of the cells with the DUSP1 inhibitor BCI-CAS 15982-84-0 followed by treprostinil (10 µM) and TGF-β1 (10 ng/mL) treatment. DUSP1 inhibition counteracted the anti-proliferative effect of treprostinil in a concentration-dependent manner, which was more effective in IPF cells compared to healthy controls (Figure 6B).

## 4. Discussion

This study investigated the regulation of miR17-92 in IPF and other lung fibrotic diseases. The data shows that specific members of the miR17-92 are specifically upregulated in fibroblasts isolated from patients with IPF or other fibrotic lung diseases. Moreover, the expression control of the miR17-92 is significantly reduced in IPF-fibroblasts. A similar disease-specific lack of response to treprostinil was observed in IPF-fibroblasts for the expression of miR-21a-5p. However, treprostinil can overcome this dysregulation and block IPF cell proliferation by upregulating the Erk1/2 MAPK inhibitor DUSP1. An overview of the loss of miR17-92 and the inhibitory action of treprostinil is provided in the graphic abstract. 

IPF is a disease that cannot be well-treated and leads to premature death [3]. Therefore, new therapeutic targets have to be identified, which is only possible if the pathogenesis of IPF is better understood. In several human cancers and in healthy fibroblasts, the miR17-92 plays a role in the control of cell proliferation and remodeling [24,25,26]. Similar to our observation, the expression of miR17-92 was reduced by TGF-β1 in a mouse model [27]. The negative expression of miR17-92 by TGF-β1 can be linked to the stimulation of C/EBP-β, which is a negative regulator of miR17-92 [28]. In other conditions, the TGF-β1-dependent downregulation of, specifically, miR-19a was followed by an increased expression and activation of Erk1/2 MAPK, which stimulated fibroblast proliferation [29]. 

The miR17-92 consists of six independent members: miR-17, miR-18a, miR19a, miR-19b, miR-20a, and miR-92 [30]. The miR17-92 was mainly studied in the context of cancer cell biology. Most studies reported an increased expression of this cluster in various cancer types, which would contradict the pro-proliferative function of one of the major targets, Erk1/2 MAPK. Analyzing the target range of miR17-92 by TargetScanHuman 7.2 (www.targetscan.org, accessed on 5 November 2019) suggested that MAPKs, especially Erk1/2 and TGF-β receptors, would be downregulated. However, in most cancer types, this mechanism does not seem to function [26,31]. 

In other lung mesenchymal cells, both PDGF-BB and TGF-β1 reduced the expression of miR-19a, and thereby increased Erk1/2 MAPK activity and proliferation [20,29,32,33]. In the above-described publications on miR17-92 in IPF patients, the downregulation of miR-19a correlated with reduced lung function [15]. Reducing miR-19a upregulated Erk1/2 MAPK and thereby increased remodeling of fibroblasts [33]. In a rat model of liver fibrosis, the expression of members of the miR17-92 was significantly reduced in fibrotic tissue compared to healthy animals [34]. The observation that TGF-β1 reduced the expression of miR17-92 by upregulating C/EBP-β [12] suggests that TGF-β1 and miR17-92 control each other’s activity. In cancer cells, C/EBP-β was described as a negative regulator of the promoter controlling miR17-92 [28]. Furthermore, TGF-β upregulated alpha smooth muscle actin (α-SMA) by stimulating C/EBP-β in human alveolar epithelial cells [3,35]. Reduced expression of miR17-92 was reported in an IPF mouse model [15]. Down-regulated mir-92a was linked to increased WISP-1 expression in IPF cells [36], and miR-18-5p reduced fibrosis by targeting the TGF-β receptor II [30]. In synovial fibroblasts, miR17-92 suppressed proliferation and migration [37]. Thus, the regulation of proliferation by miR17-92 might be disease-specific and needs to be further investigated.

All the above-described modifications of miR17-92 members confirm that its downregulation leads to increased fibroblast proliferation. This function seems to be lost in fibroblasts obtained from fibrotic lung diseases. The data presented in this study further suggest that the loss of function cannot be explained by mutation in the promoter of the miR17-92, but may depend on modified C/EBP-β regulation, which was reported in cancer cells [28]. The dysregulation of C/EBP-β is further supported by the observation that the expression of C/EBP-β was lower in fibrotic fibroblasts than in control fibroblasts. Alternatively, the reduced response of the luciferase construct containing all three promoter sections ABC in IPF cells might indicate a structural modification of the full miR17-92 promoter, which we were not able to further characterize in this study.

Others reported that miR-21 is deregulated in IPF. TGF-β1 modified miR-21 expression in fibroblasts isolated from patients with lung fibrosis [38]. Furthermore, circulating miR-21 was found in the serum of patients with IPF and correlated with lung function [39,40]. In this study, we confirmed the upregulated expression of miR-21a-5p in fibroblasts isolated from IPF patients, while we did not see a significant increase in fibroblasts from other pulmonary fibrosis. In control fibroblasts, treprostinil reduced the expression of miR-21a-5p. In contrast, neither TGF-β1 nor treprostinil had any significant effect in fibroblasts obtained from IPF patients. 

Other miRs were linked to IPF pathologies, including proliferation and excessive deposition of extracellular matrix. Increased miR-448 was reported to inhibit fibroblast proliferation and collagen synthesis by targeting JNK signaling [41]. Increased miR-766-3p and miR-1254 were linked to proliferation and extracellular matrix deposition in IPF [42]. More often, reduced expression of specific miRs was linked to the loss of fibroblasts differentiation in IPF. In human IPF-derived fibroblasts, the expression of miR-30a, miR-140, miR-324-5p, and miR-630 was reduced [43,44,45]. Interestingly, it was reported that the modification of miRs and their targets do not match in IPF [46]. This study reported that miR-29a and miR-185 were downregulated in IPF and lung cancer, but their targets, collagen Type-I and DNA methyltransferase-1, were upregulated. These findings indicate that the function of miRs in IPF might be deregulated. 

Referring to the above-described role of Erk1/2 MAPK in the control of proliferation, we provide data that treprostinil inhibits TGF-β1-induced fibroblast proliferation in IPF-fibroblasts. Treprostinil achieves this effect by upregulating the expression of the Erk1/2 inhibitor DUSP1. It had been reported before that activation of DUSP1 in pulmonary fibrosis is anti-proliferative [47]. In other conditions, it had been reported that DUSPs interact specifically with Erk1/2 MAPK and inhibit its phosphorylation, and thereby cell proliferation [48]. In cardiac fibroblasts, downregulation of DUSP1 was linked to glucose-induced proliferation [49]. In neurofibromatosis Type-I and nerve tumors, activation of DUSP1 suppressed cell proliferation [50]. DUSP1 inhibition resulted in enhanced proliferation of cardiac fibroblasts in a mouse model [51]. In another mouse model for lung fibrosis, DUSP1 was activated by the proteasome inhibitor bortezomib and reduced fibroblast proliferation [52]. 

The limitations of this study are: (i) the over-expression or inhibition of specific members of the miR17-92 was not investigated in fibrotic-fibroblasts, which was mainly due to faster senescence of the fibroblasts; (ii) we were not able to further define the nature of the loss of promoter response in IPF-fibroblasts; and (iii) it cannot be excluded that Nindetanib treatment of 5 IPF patients might have affected the expression of miR17-92, because it was reported by others that inhibited the drug PDGF-BB and TGF-β1 receptor signaling in fibroblasts [53,54]. However, the chance that such an effect is maintained in isolated cells over several cell cycle rounds in expanded cells is unlikely. We would like to mention that all primary cell lines used for this study were established from tissue samples obtained from end-stage patients undergoing lung transplantation. Therefore, our findings may not reflect the pathophysiology in early stages of fibrosis. Furthermore, it should be considered that the phenotype of fibrosis can change over time [55].

In summary, the presented data indicates that the expression of several members of miR17-92 and miR-21 is disease-specifically dysregulated in pulmonary fibrotic diseases. The role of miR17-92 in IPF is opposite to that in healthy fibroblasts, and closer to what others described for cancer cells. Therefore, the inhibition of early stages of signaling cascades or specific mitogen receptors might be less effective. However, proliferation is sensitive to Erk1/2 inhibition. However, treprostinil reduced the proliferation of fibroblasts isolated from patients with pulmonary fibrotic diseases by upregulating DUSP1.

## Figures and Tables

**Table 1:** Details of IPF and interstitial fibrosis patients. SEM: standard error of mean.

Diagnosis	Gender	Age	Therapy
IPF patients	Male	50	Pirfenidone
	Male	63	Nintedanib
	Male	50	Nintedanib
	Male	65	Nintedanib
	Male	59	steroids
	Male	48	Nintedanib
	Male	61	none
	Male	65	none
	Mean ± SEM	57.6 ± 2.5	
Interstitial Fibrosis patients	Female	52	steroids
	Male	48	none
	Male	60	steroids
	Male	69	none
	Female	68	steroids
	Mean ± SEM	59.4 ± 4.9	

## Data Availability

The data will be available on request to the corresponding author.

## Supplementary Materials

The following are available online at https://www.mdpi.com/article/10.3390/cells10112836/s1, Figure S1: Regulation of the miR17-92 promoter through C/EBP-β; Table S1: Primer sequences for PCR-based promotor generation and Sanger DNA sequencing.
