# Supplementary material for: IPF-Fibroblast Erk1/2 Activity Is Independent from microRNA Cluster 17-92 but Can Be Inhibited by Treprostinil through DUSP1
Source: Cells. 2021 Oct 21;10(11):2836. doi: 10.3390/cells10112836 (PMC8616195; doi:10.3390/cells10112836)
Supplement: Supplementary file 1 [file cells-10-02836-s001.zip › Table S1 .pdf]

Table S1. Primer sequences for PCR-based promotor generation and Sanger DNA sequencing. FWD: forward; REV: reverse.

**Promoter**	**direction**	**Primers** (restriction enzymes sites **bolded**)
HindIIIPro 1	FWD	GCG**AAGCTT**GGGGAGGTCGGAAGTACTTT
SacIPro 1	REV	GCG**GAGCTCG**CGTACAAAGTTTGGGGAAC
HindIIIPro 2	FWD	GCG**AAGCTT**GTTCCCCAAACTTTGTACGC
SacIPro 2	REV	GCG**GAGCTC**CCCACAGACTATTCTTCACC
HindIIIPro3 second	FWD	GCG**AAGCTT**GGCTCGTCGTTGCAATATCAC
SacIPro3 forth	REV	GCG**GAGCTC**GAGGAAATCTTCACATCCACG
HindIIIPro 1	FWD	GCG**AAGCTT**GGGGAGGTCGGAAGTACTTT
SacIPro 2	REV	GCG**GAGCTC**CCCACAGACTATTCTTCACC
HindIIIPro 2	FWD	GCG**AAGCTT**GTTCCCCAAACTTTGTACGC
SacIPro3 second	REV	GCG**GAGCTC**GGCAATCATAACCAACCATCC
HindIIIPro 1	FWD	GCG**AAGCTT**GGGGAGGTCGGAAGTACTTT
SacIPro3 second	REV	GCG**GAGCTC**GGCAATCATAACCAACCATCC
**Sanger DNA sequencing for promoter sequences**	**direction**	**Primers**
Promoter sequence 1	FWD	GGGGAGGTCGGAAGTACTTT
Promoter sequence 1	REV	GCGTACAAAGTTTGGGGAAC
Promoter sequence 2	FWD	GTTCCCCAAACTTTGTACGC
Promoter sequence 2	REV	CCCACAGACTATTCTTCACC
Promoter sequence 3	FWD	GGTGAAGAATAGTCTGTGGG
Promoter sequence 3	REV	AAGTGGTGGCTCTTCCAATG
