# Supplementary figures and images for: IPF-Fibroblast Erk1/2 Activity Is Independent from microRNA Cluster 17-92 but Can Be Inhibited by Treprostinil through DUSP1
Source: Cells. 2021 Oct 21;10(11):2836. doi: 10.3390/cells10112836 (PMC8616195; doi:10.3390/cells10112836)

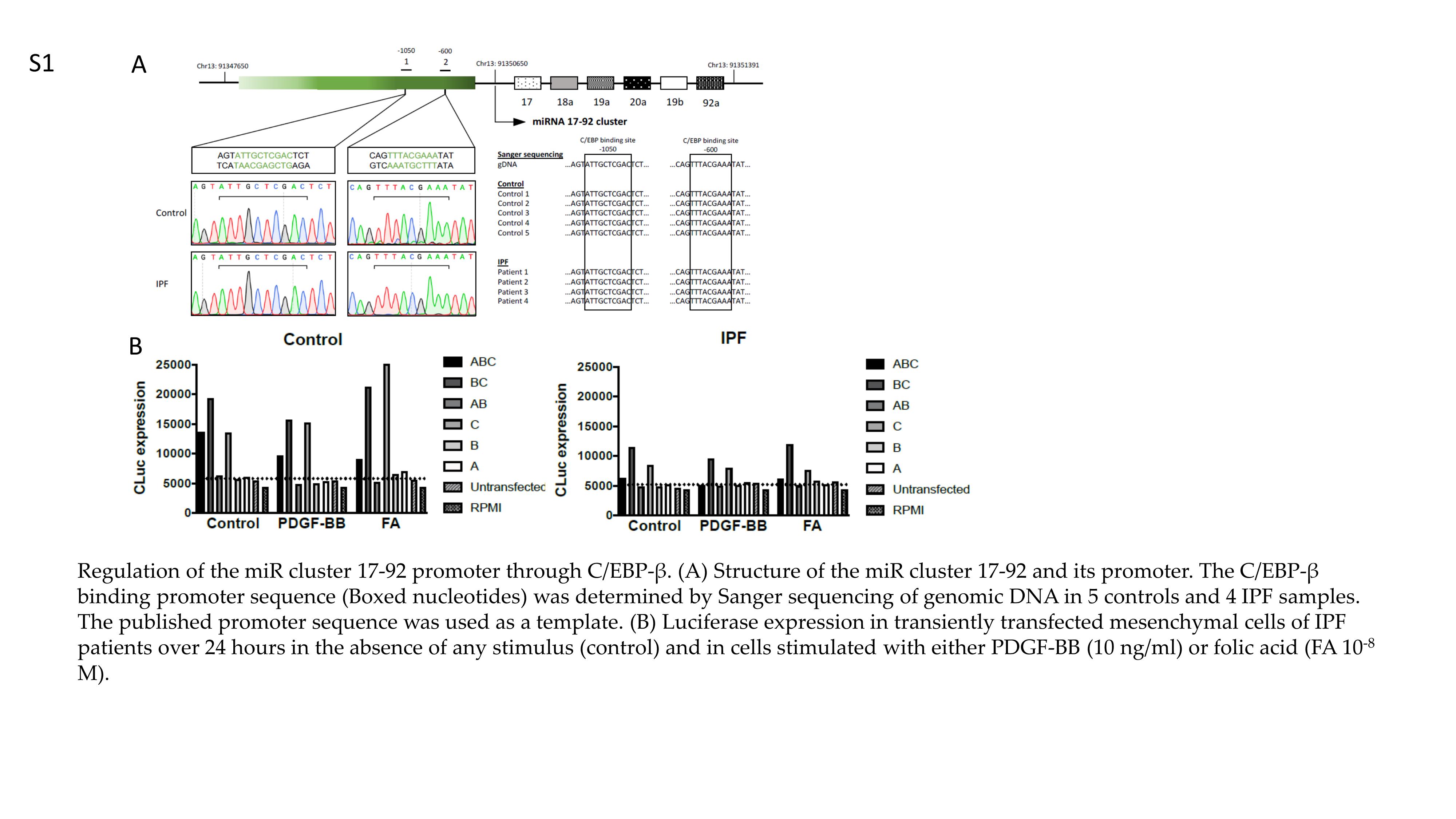

Supplement: Supplementary file 1 [file cells-10-02836-s001.zip › Figure S1.tif]
